# Supplementary material for: A novel model demonstrating that human immune cells promote multiorgan SARS-CoV-2 dissemination and human T cells limit anti-viral innate immunity
Source: Front Immunol. 2026 Oct 22;17:1951246. doi: 10.3389/fimmu.2026.1951246 (PMC13736727; doi:10.3389/fimmu.2026.1951246)
Supplement: Supplementary file 1 [file DataSheet1.pdf]

Supplementary Fig. 1

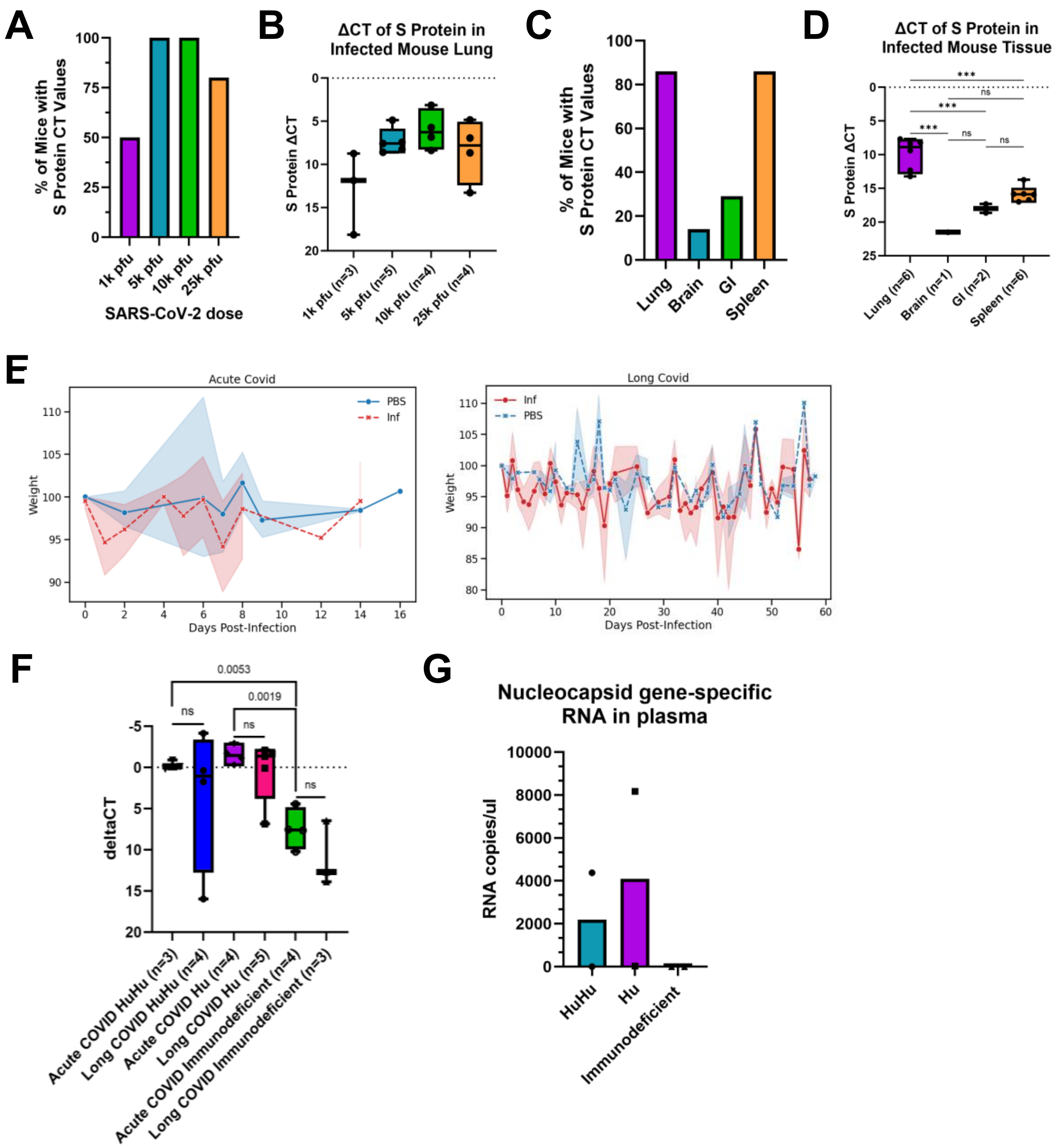

Figure S1: a. Percentage of non-humanized hACE2 KI NSG mice demonstrating SARS-CoV-2 spike protein-specific qPCR CT values intranasally infected with either 1k, 5k, 10k, or 25k pfu of WA1 at day 3 post-infection. PBS control mice (not shown) did not exhibit spike protein-specific CT values. *n*=5. **b.** Levels of viral spike protein RNA detected in the native mouse lung for all mice across all doses. ΔCT values of SARS-CoV-2 spike protein-specific qPCR were determined by normalizing the spike protein values against mouse GAPDH. There were no significant differences between groups. Box and whisker plots show min to max values. Individual values for each mouse are shown. **c.** Percentage of non-humanized hACE2 KI NSG mice intranasally infected with 5k pfu of WA1 demonstrating SARS-CoV-2 spike protein-specific qPCR CT values across multiple tissues at day 7 post-infection. PBS control mice (not shown) did not exhibit spike protein-specific CT values. *n* = 7. **d.** Levels of viral spike protein RNA detected in tissue at day 7 post-infection after infection with 5k pfu of WA1. ΔCT values of SARS-CoV-2 spike protein-specific qPCR were determined by normalizing the spike protein values against mouse GAPDH. There were no significant differences between groups. Box and whisker plots show min to max values with adjusted p-values from One Way ANOVA multiple comparisons displayed. Individual values for each mouse are shown. ***p < 0.001. **e.** Linear mixed model showing the percent weight change during the course of infection compared to baseline weight in both acute (left) and longterm (right) infection cohorts. 95% confidence intervals are shown for each group. Acute: Infected *n* = 29, Uninfected *n* = 18. Long COVID Infected *n* = 22, Uninfected *n* = 11. Infected mice in the long COVID cohort show significant weight loss compared to PBS controls. *p*-value = 0.03. There is no difference between groups in the acute infection cohort. **f.** Levels of viral spike gene-specific RNA detected in the native mouse lung for all infected mice across both acute and long-term timepoints. ΔCT values of SARS-CoV-2 spike RNA-specific qPCR were determined by normalizing the spike RNA CT values against mouse GAPDH. Individual values for each mouse are shown with *n* denoted in group name. *p*-values between groups denoted on graph, as determined by unpaired t-tests between groups. **g.** SARS-CoV-2 nucleocapsid gene-specific RNA copies per microliter, as determined by nucleocapsid gene-specific RT-qPCR using RNA isolated from plasma. Copy numbers determined using a standard curve from provided nucleocapsid standards. Hu/Hu *n* = 2, Hu *n* = 2, Immunodeficient *n* = 2.

Supplementary Fig. 2

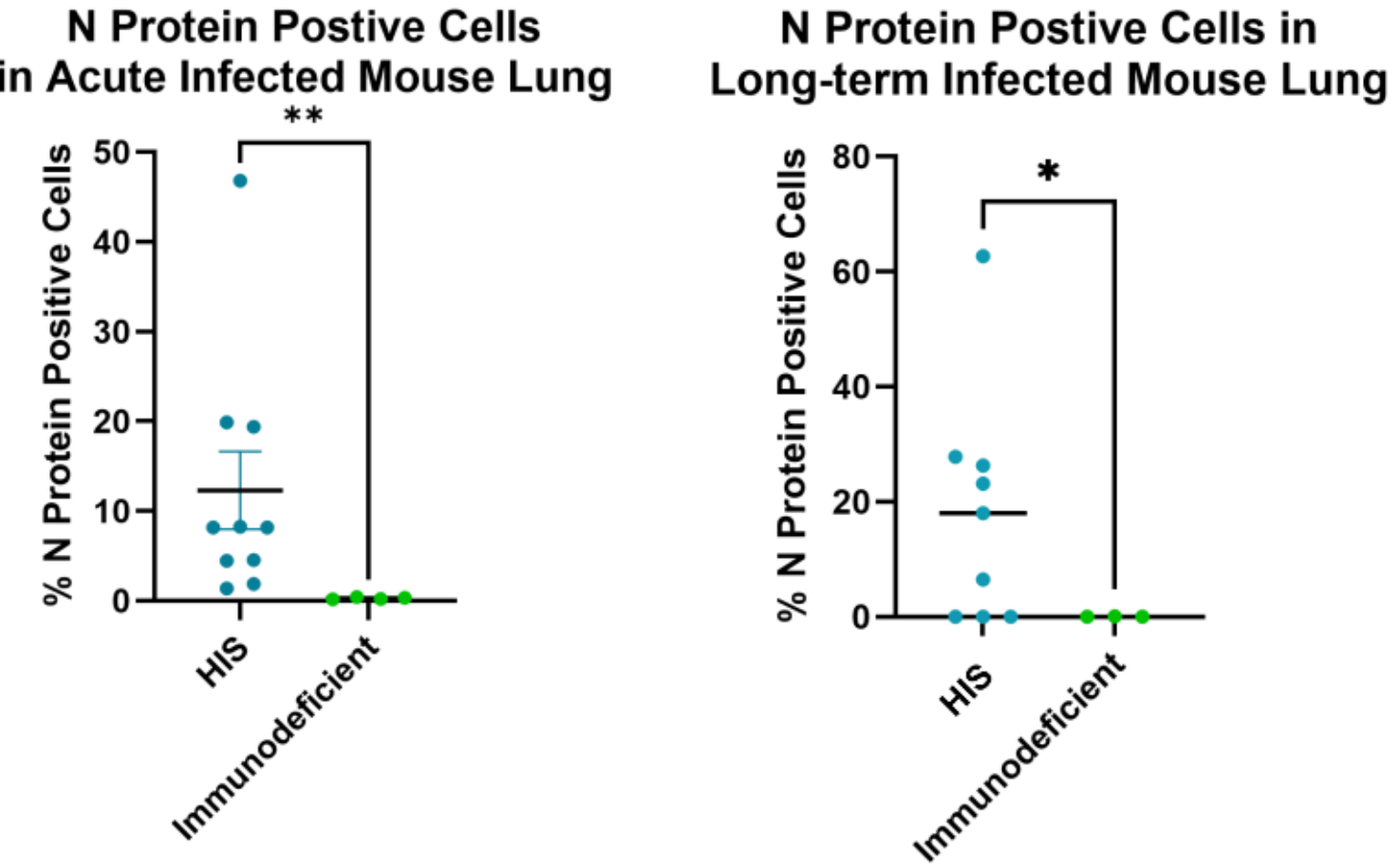

Figure S2. Percentage of SARS-CoV-2 nucleocapsid protein positive cells in infected HIS and immunodeficient mouse lung tissue calculated using HALO imaging software. Acute mouse (left) statistical significance was analyzed by Mann-Whitney test. *p*-value = 0.0020. HIS *n* = 10, Immunodeficient *n* = 4. Long-term mouse (right) statistical significance was analyzed by Welch's t test. *p*-value = 0.0272. HIS *n* = 9, Immunodeficient *n* = 3.

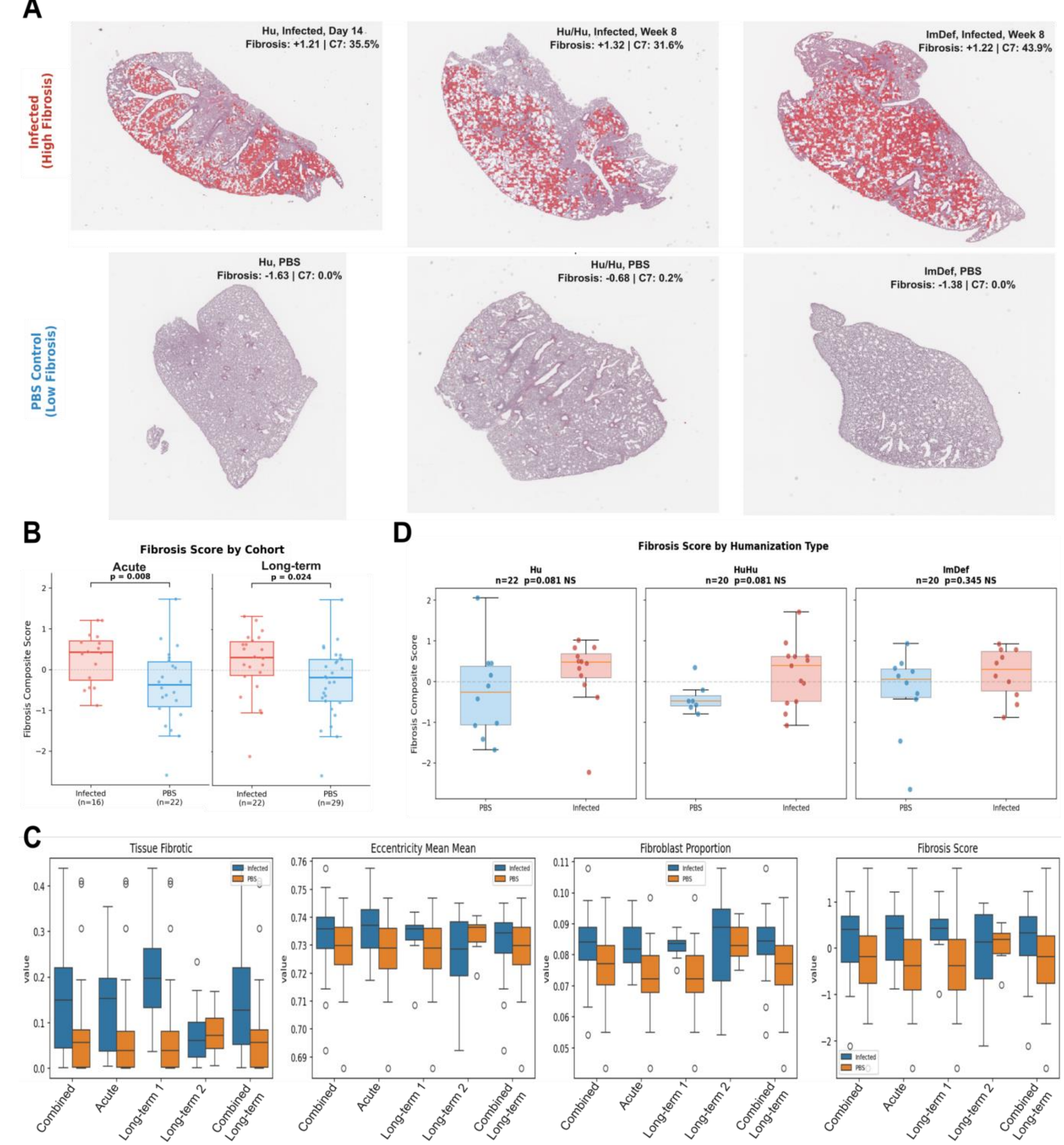

Supplementary Fig. 3

Figure S3. a. Whole-slide fibrotic cluster overlay: C7 patches (red) mapped onto representative infected (top; Hu Day14, Hu/Hu Wk8, immunodeficient Wk8) versus PBS control (bottom; Hu, Hu/Hu, immunodeficient) lung sections, demonstrating widespread fibrotic cluster distribution in infected lungs (C7 = 31.6-43.9%) compared to near-absence in controls (C7 = 0.0-0.2%). **b.** Fibrosis composite scores stratified by cohort: Acute (p = 0.008), Long-term (p = 0.024). **c.** Individual score components of the composite fibrosis score, stratified by cohort. Fibroblast proportion acute *p-value* = 0.007, tissue fibrotic proportion Long-term 1 *p-value* = 0.005 **d.** Fibrosis composite score, stratified by humanization status.

Supplementary Fig. 4

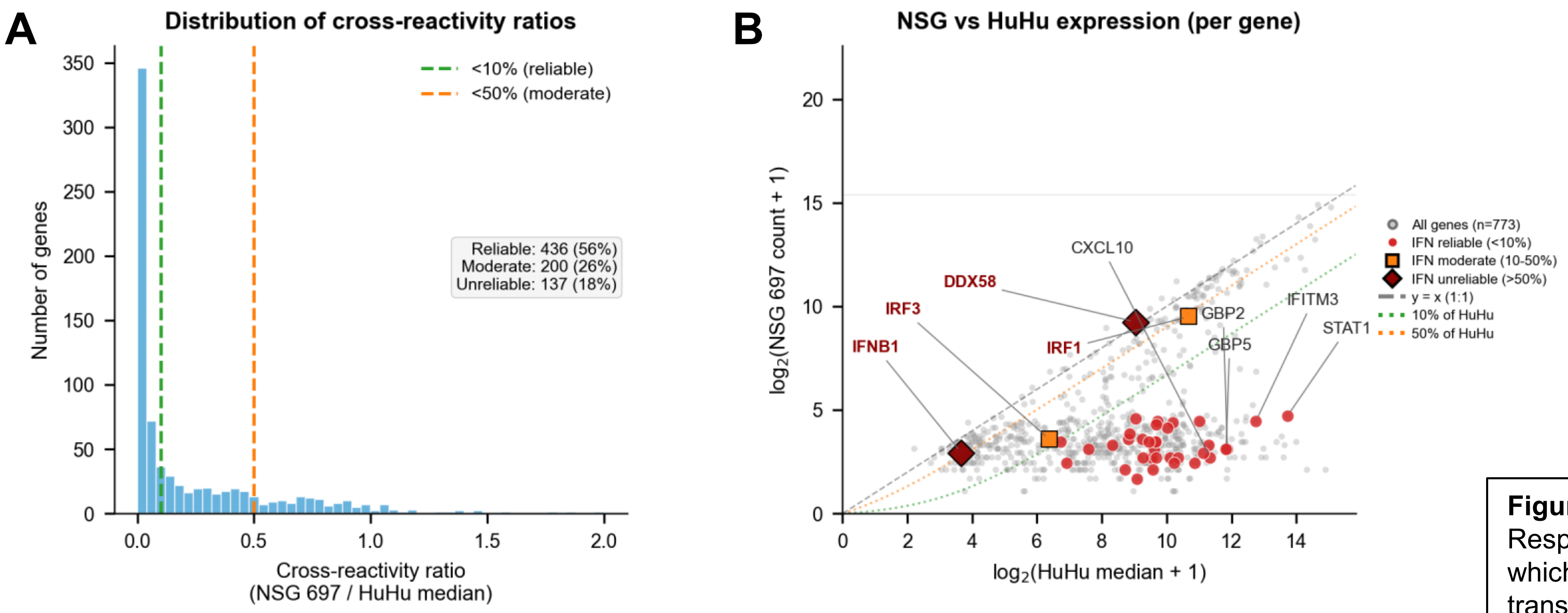

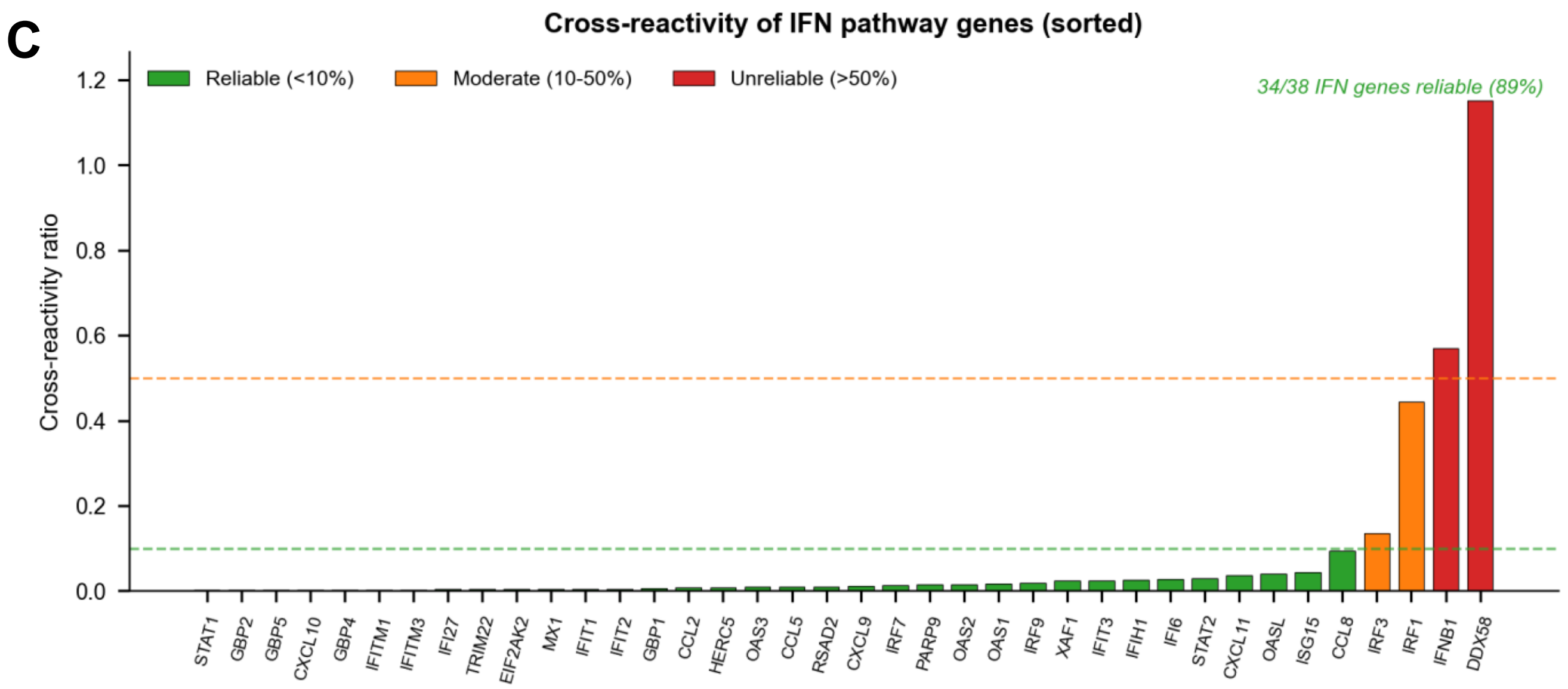

Figure S4. Cross-reactivity of the 773-gene human Nanostring nCounter Host Response panel was evaluated using an NSG control lung sample (animal 697), which lacks human immune cells and therefore produces signal only from murine transcripts that cross-hybridize to the human-targeted probes. For each gene, a cross-reactivity ratio was calculated as the positive-control-normalized count in the NSG sample divided by the median normalized count across four Hu/Hu samples. Probes were classified as *reliable (ratio < 0.10), moderate (0.10 ≤ ratio < 0.50), or unreliable (ratio ≥ 0.50).* **(a)** Distribution of cross-reactivity ratios across all 773 probes in the panel. Dashed lines indicate the 10% (green) and 50% (orange) thresholds; 436 genes (56%) were classified as reliable, 200 (26%) as moderate, and 137 (18%) as unreliable. **(b)** Per-gene comparison of $\log_2$-transformed NSG-697 counts versus the $\log_2$-transformed Hu/Hu median counts. Grey dots denote all 773 panel genes; red circles, orange squares, and dark-red diamonds highlight the 38 IFN pathway genes by reliability class. The black dashed line is the 1:1 reference (NSG = Hu/Hu); dotted green and orange lines indicate cross-reactivity ratios of 10% and 50% relative to the HuHu reference. The five most highly expressed reliable IFN genes (STAT1, IFITM3, GBP5, GBP2, CXCL10) and the four IFN genes with cross-reactivity ≥ 10% (IRF3, IRF1, IFNB1, DDX58) are labeled. **(c)** Cross-reactivity ratios for the 38 IFN pathway genes, sorted in ascending order. Bars are colored by reliability class: green (reliable, < 10%), orange (moderate, 10–50%), red (unreliable, > 50%). Thirty-four of 38 IFN genes (89%) were classified as reliable. Only three IFN genes exceeded the 50% cross-reactivity threshold (IRF1, IFNB1, DDX58); their exclusion did not alter the overall IFN pathway enrichment conclusions.

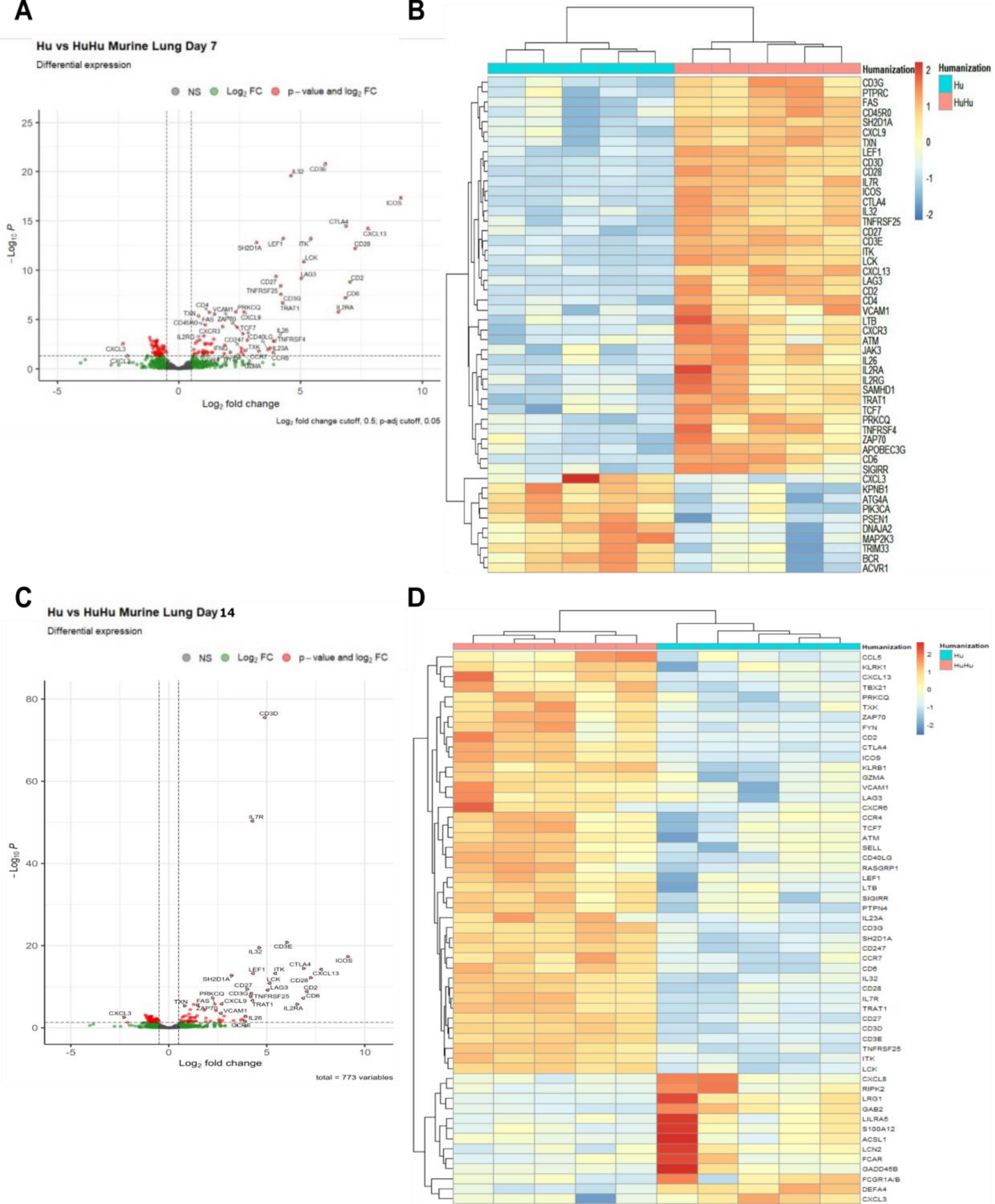

Supplementary Fig. 5

Supplementary Figure 5. a. Volcano plot of differentially expressed genes between infected Hu/Hu and Hu mouse lung samples at day 7 post-infection. **b.** Heatmap of the top 50 differentially expressed genes between Hu/Hu and Hu mouse lung samples at day 7 post-infection. **c.** Volcano plot of differentially expressed genes between infected Hu/Hu and Hu mouse lung samples at day 14 post-infection. **d.** Heatmap of all 55 differentially expressed genes between infected Hu/Hu and Hu mouse lung samples at day 14 post-infection. A *p-adj* cutoff of 0.05 is used for all differentially expressed genes.

Supplementary Fig. 6

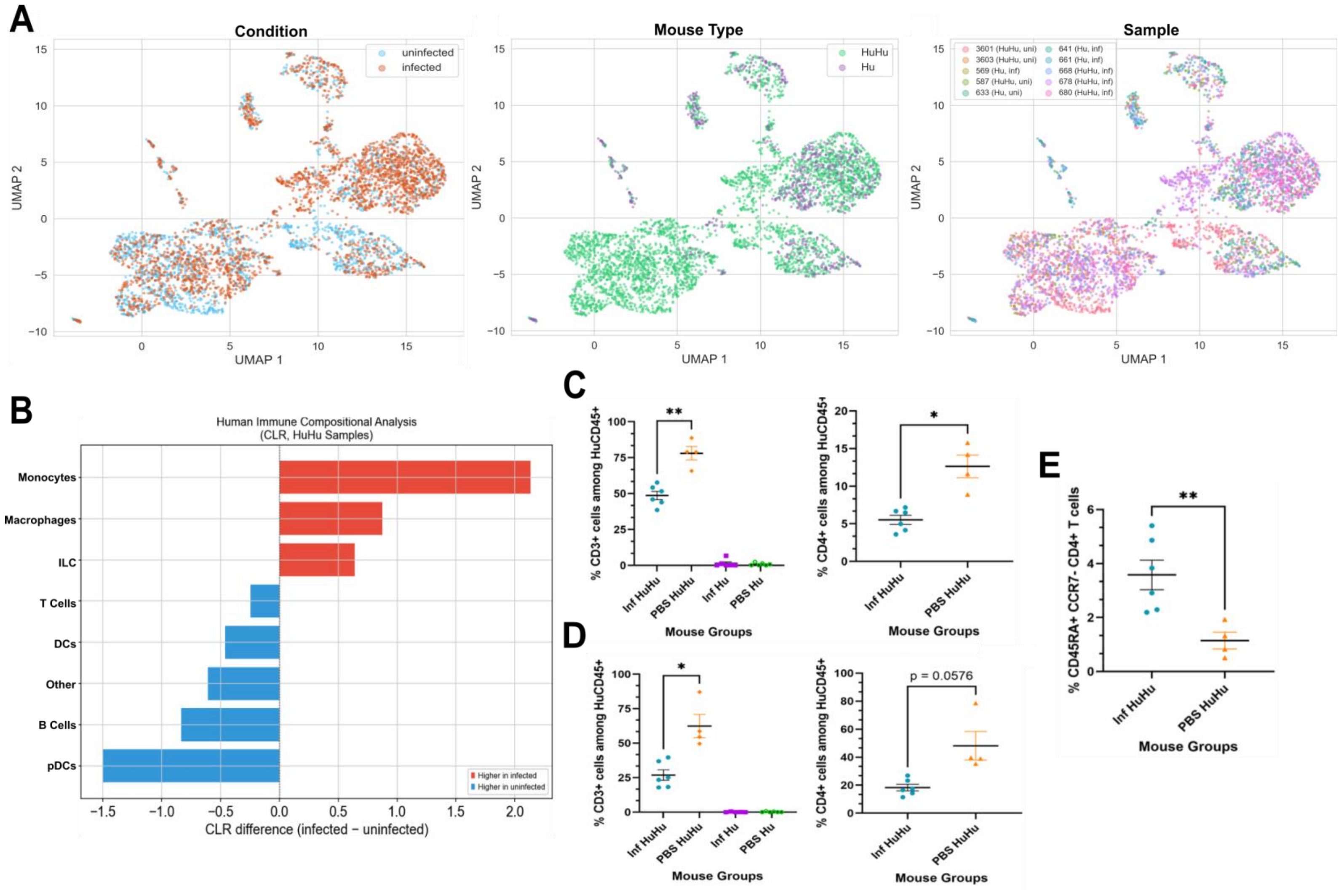

Figure S6. a. UMAP of 4,401 human immune lung cells colored by condition (left), Hu/Hu or Hu mouse (center), and sample (right). **b.** Centered log-ratio transformation of human immune cell compositional analysis of Hu/Hu samples in snRNA-seq data. Monocytes CLR p=0.1, Monocytes scCODA inclusion probability = 0.91. **c,d.** Percentage of CD3+ cells (left) and CD4+ cells (right) among all HuCD45+ cells in the mouse lung **(c)** and spleen **(d)** at 8 weeks post-infection detected via flow cytometry analysis. Infected HuHu *n* = 6, PBS HuHu *n* = 4, Infected Hu *n* = 5, PBS Hu *n* = 6. *p*-value of CD3+ cells in the lung = 0.0027 and *p*-value of CD4+ T cells in the lung = 0.0120, *p*-value of CD3+ cells in the spleen = 0.0164. All p-values determined by Welch's t-test. **e.** Percentage of CD45RA+ CCR7- TEMRA CD4+ T cells in the mouse lung at 8 weeks post-infection detected via flow cytometry analysis. Infected HuHu *n* = 6, PBS HuHu *n* = 4. *p*-value = 0.0056, as determined by Welch's t test.

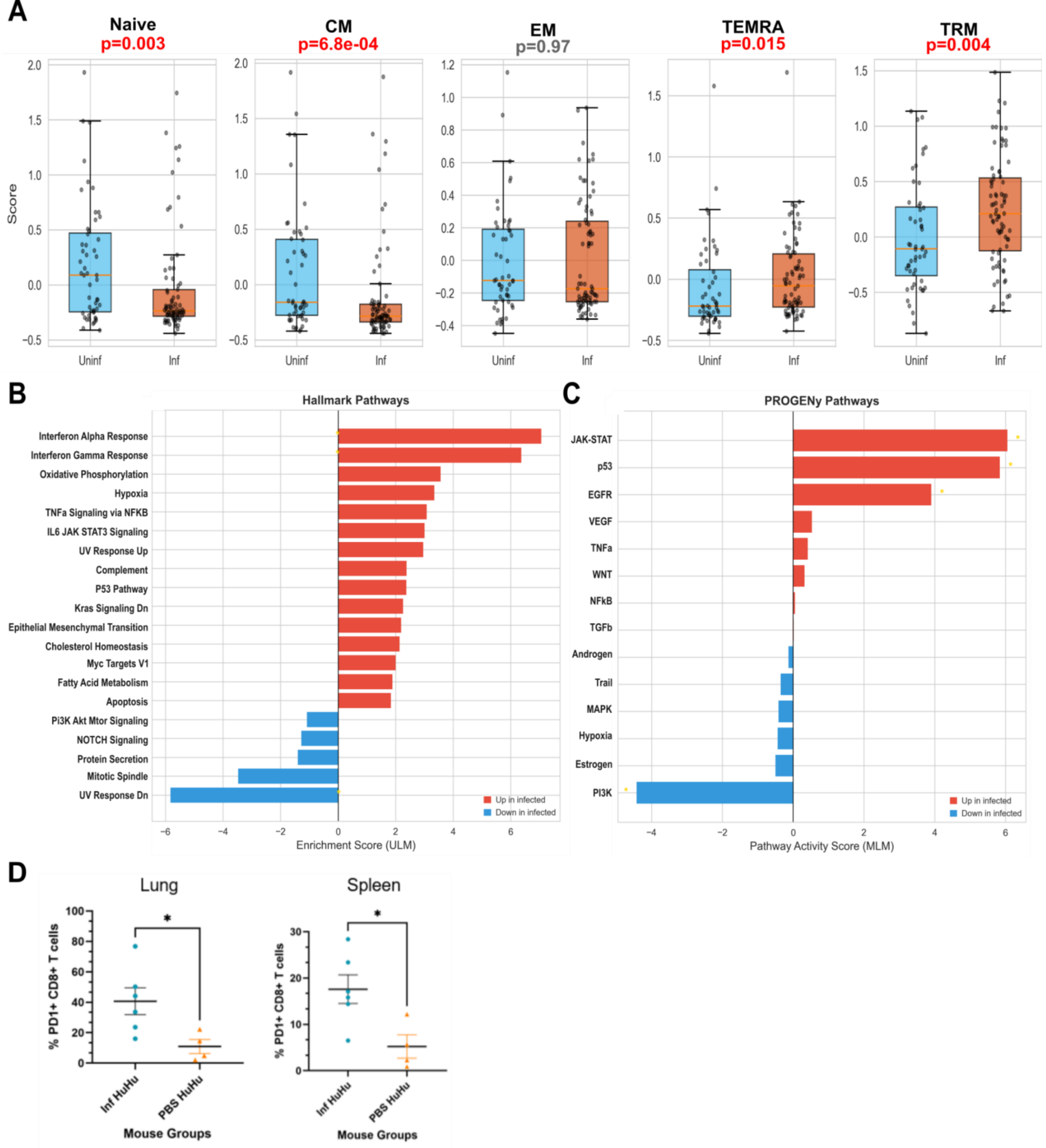

Supplementary Fig. 7

Figure S7. a. Gene-set scores computed by `scanpy.tl.score\_genes` for five CD8-relevant subsets: Naive (CCR7, SELL, LEF1, TCF7, CD27, IL7R), central memory/CM (CCR7, SELL, TCF7, IL7R), effector memory/EM (GZMK, CCL5, EOMES, IL7R), TEMRA (CX3CR1, GZMB, PRF1, GZMA, KLRG1, NKG7, GNLY), and tissue-resident memory/TRM (CD69, ITGA1, CXCR6, RUNX3, ITGAE). Box plots show single-cell score distributions; individual cells overlaid as jittered points. *P*-values calculated by Mann-Whitney U test (two-sided). Four of five subsets showed significant differences: Naive ($p = 3.0 \times 10^{-3}$, down in infected), CM ($p = 6.8 \times 10^{-4}$, down), TEMRA ($p = 1.5 \times 10^{-2}$, up), and TRM ($p = 4.2 \times 10^{-3}$, up). EM were unchanged. **b.** MSigDB Hallmark pathway enrichment scores computed by univariate linear model (ULM, decoupler) on Wilcoxon rank-sum z-scores for autosomal genes. IFN-alpha response and IFN-gamma response are the top two up-regulated pathways (gold asterisks indicate Bonferroni-adjusted $p < 0.05$). **c.** PROGENy pathway activity scores computed by multivariate linear model (MLM, decoupler). **d.** Percentage of PD1+ CD8+ T cells in the mouse lung (left) and spleen (right) at 8 weeks post-infection detected via flow cytometry analysis. Infected HuHu $n = 6$, PBS HuHu $n = 4$. *p*-value in lung = 0.0199 and in spleen = 0.0149 as determined by Welch's t test.

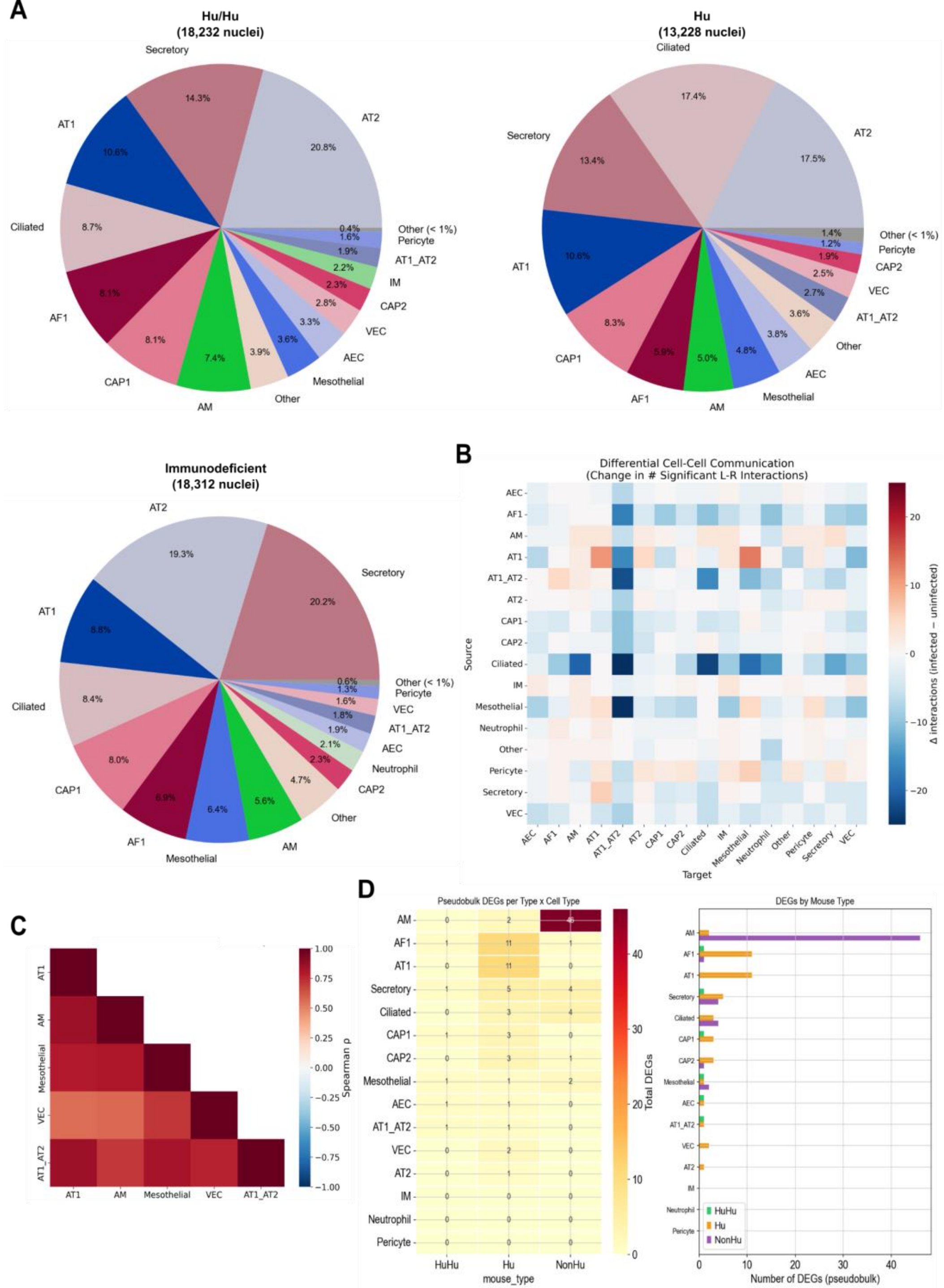

Supplementary Fig. 8

Supplementary Figure 8. a. Pie charts showing the proportion of cell populations in Hu/Hu, Hu and immunodeficient mouse lung samples as determined by clustering of snRNA-seq data. Number of nuclei detected in each group denoted above each pie chart. **b.** LIANA ligand-receptor analysis between infected cell populations. Change in interaction with infected status denoted by heatmap. **c.** Spearman correlation of DE-derived infection response scores across cell types in infected mouse lung. **d.** Pseudobulk DE results stratified by mouse type (HuHu, Hu, NonHu).

Supplementary Fig. 9

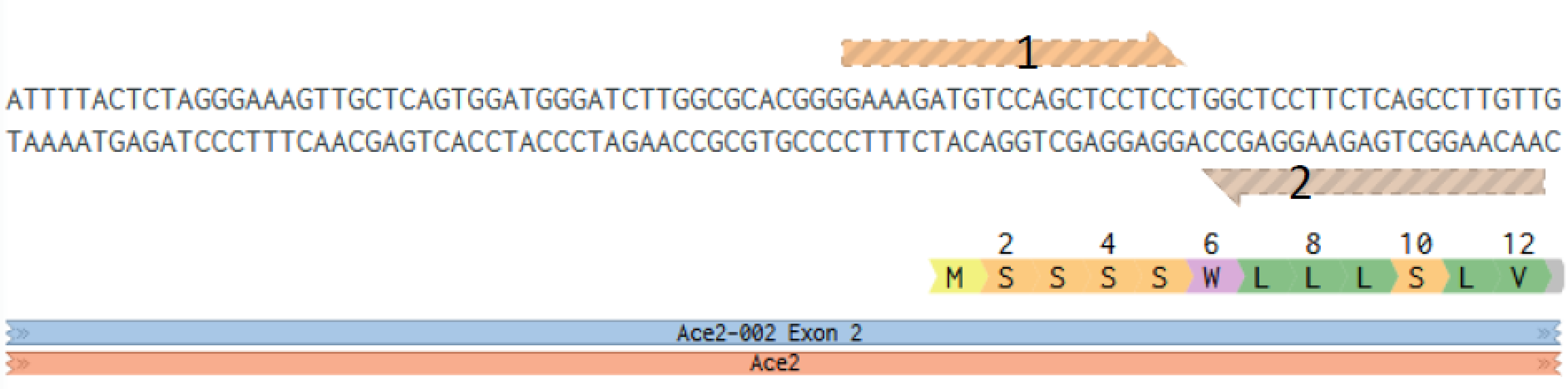

<u>Guides</u>

Ace2_Ex2_crRNA1: GAAAGATGTCCAGCTCCTCC

Ace2_Ex2_crRNA2: AACAAGGCTGAGAAGGAGCC

Supplementary Figure 9. Representative schematic for the insertion of the human ACE2 gene via CRISPR/Cas9 using guide RNAs.

Supplementary Table 1:

Day 7 Lung
Infected vs Uninfected

Pathway	P-value	Enrichment Score	NES	leadingEdge	Gene Ratio
PYRIN INFLAMMASOME SIGNALING PATHWAY	0.03345725	0.77830032	1.58102818	IL1B, PYCARD, CASP1, IL18	0.8
TCAPOPTOSIS PATHWAY	0.03325942	-0.61608009	-1.58012418	CCR5, CD3G, FASLG, CD28, CD3D, CD3E, CD247	0.77777778
THELPER PATHWAY	0.03267974	-0.600225	-1.59879767	CD3G, ITGB2, PTPRC, CD28, CD3D, CD3E, CD247, CD2, CD4	0.75
TGF BETA SIGNALING PATHWAY	0.01079914	-0.74934726	-1.66080158	TGFB3, TGFBR2, SMAD4, TGFB2, SMAD3, TGFB1	0.75
NLRP3 INFLAMMASOME SIGNALING PATHWAY	0.03339518	0.72972975	1.58028468	IL1B, PYCARD, CASP1, IL18	0.66666667
IFNG PATHWAY	0.04460967	0.76177039	1.54744952	IFNGR2, STAT1, JAK2, JAK1	0.66666667
MSP PATHWAY	0.01923077	0.8359375	1.57018903	CCL2, IL1B, TNF, CSF1	0.66666667
TOB1 PATHWAY	0.00221729	-0.64741607	-1.91687921	TGFB3, TGFBR2, SMAD4, TGFB2, CD3G, SMAD3, CD28, TGFB1, IL4, CD3D, CD3E, CD247	0.63157895
CD40 NFKB SIGNALING PATHWAY	0.01879699	0.73892523	1.66187982	NFKB2, RELB, TRAF2, CD40, CHUK	0.625
TCYTOTOXIC PATHWAY	0.03703704	-0.59694835	-1.59006977	CD3G, ITGB2, PTPRC, CD28, CD3D, CD3E, CD247	0.58333333

Table S1: GSEA results for infected and uninfected lung samples at day 7 post-infection.

Supplementary Table 2:

Day 14 Lung
Infected vs Uninfected

Pathway	P-value	Enrichment Score	NES	leadingEdge	Gene Ratio
MSP PATHWAY	0.00555556	0.89596879	1.7091761	CCL2, CSF1, TNF, IL1B	0.66666667
IFNG PATHWAY	0.01272727	0.80054854	1.62699337	IFNGR2, JAK2, IFNG, STAT1	0.66666667
CD40 NFKB SIGNALING PATHWAY	0.04667864	0.67000775	1.52566354	RELB, CHUK, NFKB2, TRAF3, TRAF2	0.625
TNFSF10 RIPK1_3 SIGNALING PATHWAY	0.00555556	0.89066111	1.69905101	TNFSF10, RIPK3, RIPK1	0.6
SHIGELLA IPAH7.8 TO NLRP3 INFLAMMASOME SIGNALING PATHWAY	0.04814815	0.78020399	1.4883398	IL1B, NLRP3, CASP1	0.6
LTF DANGER SIGNAL RESPONSE PATHWAY	0.01315789	0.6048349	1.76108625	TLR2, LTF, CD14, TLR4, TNF, IL1B, CXCL8, TRAF6, MYD88, IFNB1, IRAK1	0.57894737
APOE AND MIR146 IN INFLAMMATION AND ATHEROSCLEROSIS	0.00897666	0.7651381	1.74228329	TLR2, TLR4, TRAF6, NFKB2, IRAK1	0.55555556
TOLLLIKE RECEPTOR SIGNALING RELATED TO MYD88	0.00470958	0.54706942	1.83662686	TLR2, RELB, REL, TLR1, TLR4, TLR8, TBK1, CHUK, TRAF6, MYD88, NFKB2, TLR3, IRAK1, TOLLIP, TLR6, TRAF3	0.51612903
ERK RSK SIGNALING	0.0083682	-0.93766234	-1.66275226	MAPK1, RPS6KA1, RPS6KA3	0.5
MODULATION OF HOST RESPONSES BY IFN STIMULATED GENES	0.00166945	0.76384247	2.10131741	IFI27, IFI44, HERC5, IFIH1, IFI6, ISG15, CHUK, ATF6, TRIM25	0.47368421

Table S2: GSEA results for infected and uninfected lung samples at day 14 post-infection.

Supplementary Table 3:

Day 7 Lung
Hu/Hu vs Hu

Pathway	P-value	Enrichment Score	NES	leadingEdge	Gene Ratio
ALLOGRAFT REJECTION	0.00119332	0.65814998	2.27177994	CD3D, CD3E, CXCL13, ITK, CD28, LCK, CD2, CD3G, TRAT1, IL2RA, CXCL9, CD4, FAS, CXCR3, ZAP70, IL2RG, PTPRC, LTB, CD247, CD40LG, IFNAR2, IFNG, IL16, CD40, CD80, LCP2, IL4R, GZMA, SOCS1, HLA-A, CCR5, IL12B, CCL19, PSMB10, TAP2, CCL5, ITGAL, IL27RA, TAP1, STAT1, IKBKB, WAS, GZMB, HLA-E, IL2, IL2RB, BCL3, GBP2, STAT4, C2, IL10	0.255
TGF BETA SIGNALING	0.00520833	-0.75674966	-1.94578969	ACVR1, TRIM33, THBS1, CDH1, SMAD3, FURIN	0.11111111
ANDROGEN RESPONSE	0.01975309	-0.69939124	-1.70986152	HPGD, RPS6KA3, AKT1, MAF, NGLY1, TMPRSS2	0.05940594
ANGIOGENESIS	0.00865801	-0.92482358	-1.6043556	APP, VEGFA	0.05555556
REACTIVE OXYGEN SPECIES PATHWAY	0.04513889	0.78964129	1.52131625	TXN, SOD1	0.04081633
HYPOXIA	0.00879765	-0.58574213	-1.74414219	VEGFA, PRKCA, TGFB3, DDIT3, BCL2, JUN, RBPJ	0.035
HEME METABOLISM	0.02637363	-0.8342369	-1.56411563	MAP2K3, ATG4A	0.01

Table S3: GSEA results for infected Hu/Hu and Hu lung samples at day 7 post-infection.

Supplementary Table 4:

Day 14 Lung
Hu/Hu vs Hu

Pathway	P-value	Enrichment Score	NES	leadingEdge	Gene Ratio
IL6 JAK STAT3 SIGNALING	0.04497354	-0.38068673	-1.38178716	EBI3, CXCL3, MAP3K8, CD14, IL1R2, TLR2, CXCL10, IL1R1, CD38, CSF2RB, CCL7, IL6, CSF3R, IL1B, HMOX1, LTBR, IL15RA, CXCL11, MYD88, IRF1, IL17RA, CCR1, SOCS3, TNF, JUN, STAT3, IFNGR2, CD44, TGFB1, IL3RA, CSF1, SOCS1	0.36781609
INTERFERON GAMMA RESPONSE	0.00315457	-0.41312795	-1.63334762	CCL2, SOD2, RIPK2, IFIT2, IFIT3, IFITM3, FPR1, CD274, MT2A, IFI27, CD86, CXCL10, TNFSF10, PTGS2, NAMPT, RSAD2, CD38, CSF2RB, CCL7, CD69, ZBP1, IL6, IFI44, IL15RA, ISG15, CXCL11, MYD88, OASL, IRF1, IL10RA, PELI1, CASP1, TXNIP, IL15, TRIM25, IFIH1, SOCS3, OAS3, GBP4, JAK2, IRF4, STAT3, APOL6	0.215
INTERFERON ALPHA RESPONSE	0.03422983	-0.41708159	-1.44439049	RIPK2, IFIT2, IFIT3, IFITM3, IFI27, CXCL10, RSAD2, IFI44, CCRL2, ISG15, CXCL11, OASL, IRF1, CASP1, TXNIP, IL15, TRIM25, HLA-C, IFIH1, GBP4	0.20618557
INFLAMMATORY RESPONSE	0.01812689	-0.36682123	-1.41555341	CXCL8, CCL2, RIPK2, EBI3, C5AR1, FPR1, CD14, NLRP3, LYN, MARCO, TLR2, OSM, CXCL10, TNFSF10, MEFV, NAMPT, PLAUR, IL1R1, CCL7, CD69, IL6, AHR, IL10, RHOG, ICOSLG, CCRL2, CSF3R, IL18RAP, TLR1, IL1B, IL18, IL15RA, CXCL11, NOD2, IRF1, IL10RA, TLR3, IL15, C3AR1	0.195
TNFA SIGNALING VIA NFKB	0.01139601	-0.41691043	-1.57433228	CCL2, SOD2, RIPK2, IFIT2, GADD45B, CXCL3, CXCL2, MAP3K8, SLC2A3, TLR2, BCL3, CXCL10, PTGS2, NAMPT, PLAUR, PFKFB3, PLEK, BCL6, CD69, IL6, JUNB, ICOSLG, REL, CCRL2, IL1B, IL18, IL15RA, CXCL11, IRF1, IFIH1, SOCS3, MARCKS, TNF, MCL1, JUN	0.175
ALLOGRAFT REJECTION	0.00142248	0.52202955	1.82345775	CD3D, CD3E, TRAT1, CD3G, ITK, LCK, CD247, CD28, CXCL13, ZAP70, CD2, GZMA, CD40LG, LTB, CCL5, IL2RG, IL2RA, CXCR3, CD4, CD8B, CCR5, IL2RB, PTPRC	0.115
CHOLESTEROL HOMEOSTASIS	0.03065134	-0.80939948	-1.47448083	CXCL16, PLAUR, LGALS3, FBXO6	0.05405405
WNT BETA CATENIN SIGNALING	0.04742268	0.79202126	1.52904894	LEF1, TCF7	0.04761905

Table S4: GSEA results for infected Hu/Hu and Hu lung samples at day 14 post-infection.

Supplementary Table 5:

Acute Heart
Infected vs Uninfected

Pathway	P-value	Enrichment Score	NES	leadingEdge	Gene Ratio
IFNG PATHWAY	0.03609023	0.77719528	1.48502176	JAK2, IFNG, JAK1, STAT1, IFNGR2	0.83333333
ICOSLG ICOS PI3K SIGNALING PATHWAY	0.03618421	-0.70194733	-1.72781367	AKT2, ICOSLG, PIK3CD, AKT1, PIK3R3, ICOS	0.66666667
TYPE I INTERFERON INDUCTION AND SIGNALING DURING SARSCOV2 INFECTION	0.0129717	0.56596968	1.656771	TBK1, OAS3, TRAF3, EIF2AK2, MAVS, MYD88, OAS2, JAK1, IFNAR1, STAT1, IRF7, IRAK4, TRAF6, TLR7, IFIH1, TLR9, IFNAR2, TLR2, TLR6, TLR4	0.64516129
MYD88 DISTINCT INPUTOUTPUT PATHWAY	0.04198473	0.58094519	1.5036279	TIFA, MYD88, TLR5, TRAF6, TLR7, NFKB1, TLR9, TLR2, TLR6, TLR1, TLR4	0.61111111
TCAPOPTOSIS PATHWAY	0.04285714	-0.61333489	-1.65885868	FASLG, CD28, CD4, CD3D, CD3E	0.55555556
TOLLLIKE RECEPTOR SIGNALING RELATED TO MYD88	0.0495283	0.49892085	1.460498	REL, TBK1, TRAF3, MYD88, RELB, TLR5, IRF7, IRAK4, CHUK, TRAF6, TLR7, NFKB1, TLR9, TLR2, TLR6, TLR1, TLR4	0.5483871
IL10 PATHWAY	0.01072386	0.70190637	1.6605533	IL6, STAT5A, JAK1, IL10RA, HMOX1, TNF, STAT1	0.53846154
TLR5 NFKB SIGNALING PATHWAY	0.03689567	0.58978673	1.52651197	IL6, IL12B, MYD88, TNF, TLR5, IRAK4, CHUK, TRAF6, NFKB1	0.52941176
REGULATION OF IFNG SIGNALING	0.03129445	0.70446656	1.53401529	JAK2, IFNG, JAK1, PTPN6, STAT1, IFNGR2, SOCS3	0.5
ERK RSK SIGNALING	0.00773196	-0.95555556	-1.71909283	MAPK1, RPS6KA1, RPS6KA3	0.5

Table S5: GSEA results for infected and uninfected acute heart samples.

Supplementary Table 6:

Long-term Heart
Infected vs Uninfected

Pathway	P-value	Enrichment Score	NES	leadingEdge	Gene Ratio
YERSINIA YOPP J TO TLR2 4 MAPK SIGNALING PATHWAY	0.01724138	-0.85135135	-1.79404062	MAP2K3, MAP2K2, MAP2K4, MAP2K7	0.66666667
AML1 ETO FUSION TO PU.1 MEDIATED TRANSCRIPTION	0.0152207	0.90418354	1.49472877	SPI1, ITGAM, CD14	0.6
NLRC4 INFLAMMASOME SIGNALING PATHWAY	0.03929539	0.76920069	1.45743193	CASP1, IL1B, NLRC4	0.6
MUTATION CAUSED ABERRANT HTT TO TNF JNK SIGNALING PATHWAY	0.04927536	-0.85020243	-1.58425233	MAPK9, MAPK8, MAP2K7	0.5
MUTATION ACTIVATED KRAS NRAS TO ERK SIGNALING PATHWAY	0.01515152	-0.78213802	-1.7872891	MAP2K2, KRAS, MAPK1, NRAS, RAF1	0.5
LTF DANGER SIGNAL RESPONSE PATHWAY	0.02050114	0.62542656	1.54534316	TRAF6, IL1B, TLR2, NFKB1, IRAK4, CD14, TNF, IL6, LTF	0.47368421
TRK FUSION KINASE TO RAS ERK SIGNALING PATHWAY	0.01515152	-0.78213802	-1.7872891	MAP2K2, KRAS, MAPK1, NRAS, RAF1	0.45454545
UPTAKE AND FUNCTION OF ANTHRAX TOXINS	0.03333333	-0.7061793	-1.72857989	MAP2K3, MAP2K2, MAP2K4, MAP2K7, CALM1	0.45454545
RET FUSION KINASE TO RAS ERK SIGNALING PATHWAY	0.01515152	-0.78213802	-1.7872891	MAP2K2, KRAS, MAPK1, NRAS, RAF1	0.45454545
MACROPHAGE MARKERS	0.00406504	0.86851193	1.64560047	CD163, RAC2, CD14, CD68	0.44444444

Table S6: GSEA results for infected and uninfected long-term heart samples.

Supplementary Table 7:

Acute Brain
Infected vs Uninfected

Pathway	P-value	Enrichment Score	NES	leadingEdge	Gene Ratio
IL22BP PATHWAY	0.03819444	0.65290988	1.55550048	JAK3, JAK1, SOCS3, JAK2, TYK2, STAT1, STAT3, STAT5A	0.88888889
IFNG PATHWAY	0.04664179	0.75333333	1.48120117	JAK1, IFNG, JAK2, STAT1, IFNGR2	0.83333333
TGF BETA SIGNALING PATHWAY	0.00458716	-0.80266078	-1.79992189	SMAD4, SMAD3, TGFB2, TGFB3, TGFBR2	0.625
TYPE I INTERFERON INDUCTION AND SIGNALING DURING SARSCOV2 INFECTION	0.00988468	0.52324882	1.6765937	JAK1, IRAK4, IFIH1, OAS1, TLR2, TLR3, OAS2, TLR9, EIF2AK2, TYK2, IRF7, TRAF6, TLR4, TRAF3, IRF9, STAT1, STAT2	0.5483871
IMMUNE RESPONSE TO TUBERCULOSIS	0.03553299	0.5429828	1.57216965	JAK1, MX1, IFITM1, OAS1, JAK2, TYK2, IFIT3, IRF9, STAT1, IFNGR2, STAT2, SOCS1	0.52173913
KSHV MIR1 2 TO ANTIGEN PROCESSING AND PRESENTATION BY MHC CLASS I MOLECULES	0.02340426	-0.82130104	-1.59537306	HLA-E, HLA-B, HLA-C	0.5
TOB1 PATHWAY	0.02970297	-0.57707501	-1.69909355	SMAD4, SMAD3, TGFB2, CD3G, IL4, TGFB3, IL2, TGFBR2, CD28	0.47368421
CO STIMULATION BY ICOS	0.02430556	0.68475652	1.63137229	ICOSLG, PIK3CB, ICOS, PIK3R6, PIK3R3	0.45454545
LUNG PATHOLOGY OF COVID19	0.00332779	0.65866368	1.89003107	TNF, CCL2, CXCL9, IFIH1, CXCL10, IL6, CXCL11, NFKB1, TLR3	0.45
CD163 MEDIATING AN ANTI-INFLAMMATORY RESPONSE	0.00559701	0.85197047	1.67514114	CD163, IL6, MAPK14, IL10	0.44444444

Table S7: GSEA results for infected and uninfected acute brain samples.

Supplementary Table 8:

Antibody name	Clone	Vendor
anti-human CD45 PE-CF594	HI30	BD Biosciences #562279
anti-mouse CD45 APC-Cy7	30-F11	BD Biosciences #557659
anti-human CCR7 BV421	G043H7	BioLegend #353208
anti-human CD3 PerCP Cy 5.5	SP34-2	BD Biosciences #552852
anti-human CD4 V500	RPA-T4	BD Biosciences #560768
anti-human CD8 AF700	RPA-T8	BD Biosciences #557945
anti-human CD14 PE	HCD14	BioLegend #325606
anti-human CD19 APC	HIB19	BioLegend #302212
anti-human CD45RA FITC	HI100	BD Biosciences #555488
anti-mouse TER119 PE-Cy7	TER-119	BioLegend #116222
Propidium iodide	N/A	Cytek Biosciences #13-6990-T200
Anti-human CD4 BUV395	SK3	BD Biosciences #563550
Anti-human CD3 BV605	UCHT1	Biolegend #300460
Anti-human CD8 BV650	SK1	Biolegend #344730
Anti-human CD123 BV711	6H6	Biolegend #306030
Anti-human CD14 BV786	M5E2	BD Biosciences #563698
Anti-human CD25 PE	BC96	Biolegend #302606
Anti-human CD11c PE Cy5	B-ly6	BD Biosciences #551077
Anti-human CCR7 PE Cy7	G043H7	Biolegend #353226

Table S8: List of antibodies used for flow cytometry analysis.

Supplementary Table 9:

Antibody name	Vendor
**anti-Sars-CoV2-nucleocapsid**	Sinobiological #40143
**anti-SARS-CoV2 spike glycoprotein**	abcam ab272504
**anti-hACE2**	abcam ab108209

Table S9: List of antibodies used for immunohistochemistry.

Supplementary Table 10:

Gene	Cross-reactivity ratio	Human signal fraction	Class
DDX58	1.150	0.000	unreliable
IFNB1	0.569	0.431	unreliable
IRF1	0.444	0.556	moderate
IRF3	0.135	0.865	moderate
CCL8	0.095	0.905	borderline reliable
All other IFN genes (33)	<0.10	>0.90	reliable

Table S10: Cross-reactivity of IFN genes.
